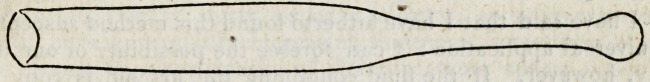# Book Reviews

**Published:** 1826-11

**Authors:** 


### CRITICAL ANALYSES.

Quæ laudanda forent, et quæ culpanda, vicissim

Illa, prius, cretâ; mox hæc, carbone, *notamus.*—Persius.

*A Comparative View of the more intimate Nature of Fever: deduced from Physiological Analysis, and illustrated by Critical Remarks and Practical Observations.* By James Black, M.D. S.R.N. and Member of the Royal College of Physicians of London.—8vo. pp. 118. London: Longman and Co. 1826.

Dr. Black has written a sensible, but we cannot flatter him so far as to call it an interesting, work. It has for its object to investigate the nature of fever in general, without reference to its particular forms; and with this view he collates and compares some of the principal theories which have at different times been propounded, and, finding them insufficient to explain the phenomena, he presents us with his own opinions on the subject.

The author begins by considering the " primary stage" of fever; and having shown, what is indeed undeniable, that the morbid change cannot have resulted from any appreciable increase or diminution of the solids or fluids, he argues further, that as, besides these, there are no other " elements" in the body, "save the different powers of mind, motion, and life, it is presumed that some lesion or affection of these imponderable essences has given rise to the elicited phenomena." The share which these have in fever is next investigated, and the first important conclusion at which we arrive is, that although the brain and nerves of involuntary motion are intimately implicated in all well-developed fevers, still, according to Dr. Black's opinion, that they are primarily affected, is not so consonant with general facts and observation.

Passing on to some other source whence the morbid phenomena in question may proceed, we are told that there is another most important power, in more immediate connexion with the matter of our bodies than either the brain or nerves, giving place only on the commencement of chemical decomposition: this he calls "muscular power, or materia vitæ;" and to this the preliminary phenomena of fever are referred. It will scarcely be expected that the impaired energies of the system are to be accounted for by any increase in this same materia vitæ, and accordingly its diminution is regarded as the first link in the chain.

"Capillary circulation is affected among the very first *mobilia* of fever; and this capillary affection consists of an impaired or a diminished action, which, it is known from strict physiology, can only happen from a decrease of the motive and vital powers of this system, where no sensible lesion has otherwise taken place. The lassitude or weariness of the muscles, where no undue exertion, nor even any, has preceded, can only be accounted for by the fibres being deprived of the healthy proportion of the materia vitæ: in fact, they are, in one sense, in the same condition as if they had been exhausted by severe exercise. The nerves of volition transmit their ineffectual stimuli to both conditions of the fibres; but, in the one case, the irrespondence excites no surprise nor uneasiness, while, in the other, we have the apparent anomaly of weariness and the pains of exhaustion, without previous exercise. A cold and shrunk skin, with the sensation of chilliness, is also referred to the partial absence of superficial vitality and to diminished capillary circulation, while the nerves are ready to transmit to the sensorium the changes taking place at their extremities; not forgetting that the more internal vessels, having also lost their tensiveness, are, in this stage, relatively overcharged with blood, and giving the sensation of pressure and of greater heat to another set of nerves; which increases the relative sensation from those arising from the surface of the body. The small and frequent pulse arises from the oppressed and diminished action of the heart, occasioned by an undue load of blood and impaired muscular power; the consequence of which is, that the heart does not fully contract and empty itself, but the imperfect systole more frequently takes place, to get rid of the same quantity of blood in the same section of time; and, besides, the capillary (reticular) circulation being almost neutralised or stopt, the blood must make its circuit in a shorter period of time, else it would become more or less congested in the vessels. To an impaired muscular power over the whole system, at least to the extent of embracing the heart in the lesion, the initial phenomena of fever are attributed; but in what exact manner this is accomplished by poisons, cold, stimuli, and contagion, it is not attempted at present to inquire. A negative condition of this power, it is inferred, may physiologically be granted to be an essential condition of the first cognisable symptoms: it is therefore taken as an ultimate fact in the present stage of pathological investigation, and it is left to other and future research to carry the solution of causation farther up the scale of nature." (P. 22.)

We have already stated that our author examines the opinions of the principal writers upon fever: among these there are two who, we suspect, will scarcely thank him for the mention he has made of them,—we mean Dr. W. Philip and Dr. Armstrong. In reference to the former, he quotes his Treatise on Febrile Diseases, published in 1797, in which he states "that the proximate cause of fever is a change in the laws of excitability, in consequence of which the same agents no longer produce the same effects:" and again—"fever may be either a state of excessive excitement or debility of all the functions, without local affection." Now this is no more than a modification of the Brownonian theory, and we think it scarcely fair to expect that a work so crude as that from which the quotation is taken, can be regarded as containing the opinions of its author at a distance of thirty years, during which time he has written other works, in which no mention is made of these distinctions of fever; a circumstance, indeed, pointed out by our author himself. With respect to Dr. Armstrong, he takes as *his* pathology of fever that which is contained in the edition of his work on Typhus, published in 1816; but there are some whose opinions do not require thirty years to undergo a revolution.

The "secondary stage" is next spoken of, and the various phenomena of reaction detailed; but in this part of the discussion we find nothing either new or interesting.

Symptomatic fever is regarded as proceeding from the same source as idiopathic, the effect of an injury being to impair, according to its extent, the muscular power. Exhaustion and expenditure of vitality are propagated by continuity or similarity of texture, by the medium of the nerves or by the blood, to the heart, or even to the brain, by which the vascular system is brought into this negative state with regard to its muscular power; and hence the series of febrile actions. "The induction of symptomatic fever would, however, appear to require only the heart and blood-vessels to be reduced in their materia vitæ, as the brain is not commonly much affected."

The doctrine of "irritation" occupies some share of our author's attention, and leads to various quotations from the modern French writers, but to nothing satisfactory. After some cursory remarks upon the coagulation of the blood, as connected with the form of fever called inflammatory, the author comes to the following conclusions, which may be regarded as containing a summary of his peculiar views.

"First. A fever essentially and primarily consists in a negative state of the muscular power, as compared with the physical organism or substratum of the body; and it appears that it is sufficient to constitute the febrile act, for the heart and blood-vessels to be alone involved in this relative condition.

"Secondly. This negative state may be produced, either by exhaustion, as from heat, violent exercise, or stimuli; or it may be the direct or instantaneous result of an injury, or of the application of debilitating powers,—as of some poisons and contagions.

"Thirdly. A stage of atony, torpor, or collapse, however short, and sometimes obscure, but at least to embrace the heart and blood-vessels, is the initial condition of every symptomatic and inflammatory fever, as well as of those of the lowest and most contagious type.

"Fourthly. This negative state of the muscular power is, in the first instance, either quickly followed by death, or, sooner or later, by increased frequency or quickness of the pulse, arising from the stimulus and pressure of the increased volume of blood on the heart, now relatively reduced in its vitality. This frequency of the heart's action is also promoted by accumulating heat, the consequence of repressed secretion and perspiration, and by the sensation of pain or irritation being transmitted through the nerves to the heart, or sensorium, or to both; thus exhausting the muscular power, and increasing the mobility of the moving fibre.

"Fifthly. All fevers, commonly so called, are the constituted sequences of a prior condition: they are strictly physiological reactions.

"Sixthly. The reaction in any case will be as the previous strength and plethora of the constitution, joined to the quickness and the degree to which the materia vitæ has been affected, provided it has not suffered to an immediate irretrievable extent: and the mildness of a formed fever will be proportioned to the small extent to which the muscular power has been injured; while the lowness and putridity of the type will again depend on the depraved or reduced state of the body previous to the disease, added to the greater or less universal lesion of the muscular power.

"Seventhly. A fever will recur and continue, at least until the *inertia* and bulk of the solids and fluids of the body be brought on a par with the powers of the muscular life, either by the reduction of the former, or by the increase of the latter; which equilibrium not being obtained, dissolution must follow.

"Lastly. The exact causes of idiopathic fevers, assuming the different periodical types, or being continued, are obscure; as we are, in one regard, ignorant of the nature of the exciting causes, beyond perceiving that they have generically a debilitating effect on the muscular principle.

We dissent from the first position: it appears to us that too much stress is laid throughout upon the state of the muscular system; neither do we think it "sufficient to constitute the febrile act, for the heart and blood-vessels to be alone involved in the relative condition." We regard fever as essentially consisting in an impression made upon the brain and nerves, the nature of which we do not, and probably never shall, know: while, with regard to the subsequent phenomena, they are as general as is the influence of that system, on the primary derangement of which they depend. Sometimes one part is more peculiarly involved, sometimes another: but we agree with Dr. Fordyce, who, speaking of fever, says, "it is a disease that affects the whole system; it affects the head, the trunk of the body, and the extremities; it affects the circulation, the absorption, and the nervous system; it affects the skin, the muscular fibres, and the membranes; it affects the body, and it affects likewise the mind."

These theoretical views of fever are followed by some observations on the various indications of cure, and the individual remedies to be employed in their fulfilment; but this part contains nothing of sufficient importance for quotation.

We stated at the commencement of the present article, that the work was not very interesting: this appears to us to depend, in a great measure, upon the involved and somewhat pedantic style adopted by the author. Thus, speaking of those who refer fever to a local cause, he says, "When once inflammation was detected in the progress of a fever, it was immediately made the co-efficient to the whole equation of phenomena, which then received their separate values, and the solution of the whole was supposed to be satisfactorily elicited." (P. 11.) Other examples of a similar nature might be given, but the above is sufficient to illustrate our meaning.

---

*Transactions of the Medico-Chirurgical Society of Edinburgh.* Vol. II. With Plates.—8vo. pp. 411. Black, Edinburgh; Longman and Co. London. 1826.

(Concluded from page 363.)

*An Account of several Cases of Poisoning with Arsenic.*
By Dr. Christison.

The object of the very intelligent author, in the first part of this paper, is to show that the liquid re-agents, usually employed in the detection of arsenic, may be altogether superseded by the method of reduction and sublimation, which he suggests may be rendered so delicate as to afford more minute evidence than any liquid test. When the student sets about repeating the experiments described by chemists as calculated to detect the presence of arsenic, he invariably (or almost invariably) employs distilled water; and nothing, certainly, can be more clear and satisfactory than the results thus obtained. But it is quite obvious that this kind of investigation is of little avail when we are called upon to decide in cases of poisoning, where either the metal is found in such quantity as scarcely to require the assistance of any chemical manipulation, or else every step of the process is embarrassed by the alimentary and other matters contained in the stomach, interfering with the action of the tests, and obscuring the results by modifying the colour of the product; and, although various methods, especially that of Mr. Phillips, have been found, to a certain extent, efficacious in destroying the colours of these mixed fluids, yet we agree with Dr. Christison that none of them can be safely applied to medico-legal investigations. The manner in which our author recommends the examination to be conducted, will best appear from the following account of an analysis performed by him three months after the death of an individual, supposed to have been poisoned. Part of the contents of the stomach had been preserved, and the details are thus given:

"The substance transmitted was a thick, slightly viscid, putrescent, dirty-gray fluid, with a few floating coagula; and it amounted to an ounce and a quarter. It was poured into a porcelain vessel, diluted with the washings of the phial, boiled briskly for fifteen minutes, and thus changed into a transparent liquid, with dirty ash-gray flocks suspended in it. The solid matter being separated by filtration, a clear fluid was obtained, of a very pale, straw-yellow colour.

"A small portion of this fluid was tested with lime-water, and the ammoniacal nitrate of silver. Lime-water caused a very scanty, dirty grayish-white, floculent precipitate. The ammoniacal nitrate of silver caused a copious, dirty grayish-white, pulverulent precipitate, without the slightest tint of yellow.

"As the arsenic, if present at all, evidently existed in very minute proportion, and the fluid was so composite as to render the foregoing tests inconclusive; instead of wasting any more of it upon trials with the other liquid re-agents, the whole of the remainder, with the washings of the filter, was acidulated with acetic acid, and subjected for fifteen minutes to a stream of sulphuretted hydrogen gas. It acquired, in consequence, a deeper yellow colour, and muddy appearance: which, when the excess of sulphuretted hydrogen was driven off by ebullition, gave place to a considerable precipitate, of a pale lemon-yellow colour, and composed partly of fibrous flocculi, partly of very minute, brilliant scales. The presumption that arsenic existed in the fluid was thus strong.

"Next morning, the precipitate having fallen down, the supernatant fluid was withdrawn with the pipette, and its place supplied with distilled water. The brilliant scales then became still more distinct. The operation of subsidence and affusion being repeated the same day, the precipitate was thrown upon a filter; and next morning, when the water had passed through, the filter was compressed, and partially dried between folds of bibulous paper. The moist precipitate was then removed, dried in fragments with a gentle heat, dropped into the bottom of a tube of this shape and size, and covered with a little black flux, which was dropped through a paper tube, so as not to soil the glass. The whole material just filled the ball at the end of the tube. Heat was then cautiously applied with a very small spirit-lamp flame. Some water, disengaged at first, was removed with a roll of filtering paper. On the farther application of heat, which was raised to full red, a distinct crust was sublimed into the narrow part of the tube. It was smooth, bluish-gray, and brilliant, like polished steel, externally; crystalline, sparkling, and iron-gray internally, like the fracture of fine steel.

"The portion of the tube containing the flux being removed by melting and drawing it out, the crust was driven up and down by the spirit-lamp flame. At first it kept its polish and iron-gray colour; but at length it was all converted into little detached crystals, white, translucent, of adamantine lustre, and evidently showing triangular facets under a microscope of four powers. These crystals, I need hardly add, were octaëdral crystals of oxide of arsenic. The tube was weighed before and after the crystals were washed out with distilled water; and their weight thus proved to be one-twentieth of a grain.

"The solution, amounting to a drachm, was divided into three portions, and subjected to the ammoniacal sulphate of copper, the ammoniacal nitrate of silver, and sulphuretted hydrogen. These tests all gave pointed indications; but it required very cautious management to make them distinct.

"This analysis left no doubt that the deceased had taken arsenic; and, as the symptoms and mode of death, as well as the morbid appearances, corresponded with what arsenic is known to produce, I could not hesitate to ascribe his death to its administration." (P. 289.)

Dr. Christison assumes that the arsenic in the matter transmitted to him scarcely exceeded the twentieth part of a grain; a quantity, minute as it is, which he thinks will never fail, in careful hands, to afford complete evidence.

A second case is related, the chief peculiarity of which consists in the entire absence of any morbid appearances in the stomach. This circumstance is very rare, and has only been recorded as occurring in those instances where the patients die soon after the administration of the poison, as in the present case, where the individual (a girl fourteen years old) survived only five hours. Only one difficulty is anticipated as likely, when it takes place, to impede the method proposed above: it is thus described, and the means of obviating it pointed out.

"I have said that I have hitherto found this method susceptible of universal application: I can foresee the possibility of one difficulty, however. If the fluid containing the arsenic is ropy and viscous, even after being boiled and acidulated with acetic acid, the sulphuret thrown down may be mingled with so much animal matter, that the metallic sublimate is not distinctly formed, on account of the great quantity of empyreumatic matter suddenly projected along with it. This has happened to me once only out of at least a hundred experiments. In that case, an addition must be made to the process. The criterion by which we may know that the additional process is required, is the colour of the precipitate. If the colour is lemon-yellow, or pale brownish-yellow, it is unnecessary: if it is cream-white, the simple process will probably fail. The plan I should prefer is a modification of that proposed by Berzelius, and of the method followed by Roloff, for determining the quantity of arsenic in the sulphureous precipitate. Treat the precipitate with nitric acid, and thus convert the sulphuret into sulphuric and arsenic acids; dissolve and filter; neutralise with ammonia, and throw down the arsenic and sulphuric acids with nitrate of lead; reduce the precipitate, which contains arseniate of lead, in the usual way." (P. 306.)

The second part of the paper relates to the "symptomological evidence" of poisoning by arsenic, principally in the form of a lengthened commentary on a case, in which its administration was deemed probable. Into this we cannot enter; but we may observe, that the author regards the symptoms as capable of affording more satisfactory evidence of the previous exhibition of various poisons, than writers on medical jurisprudence are generally inclined to allow.

With the following illustration of the *peculiar* effects of some poisons, (three of them common,) we conclude our account of this interesting paper.

"There are some poisons, the symptoms of whose action are generally so characteristic, that an experienced person cannot confound them with any natural disease, or with any other poison. Others possess this characteristic action more rarely. Of the first kind are *oxalic acid* and *strychnia;* of the second, *corrosive sublimate* and *arsenic.*

" Few opportunities have hitherto occurred for ascertaining correctly the symptoms of poisoning with oxalic acid, in those most frequent cases in which it proves fatal within an hour. The instances, however, which have been accurately observed, coupled with what is known of its effects on animals, lead to the conclusion that it generally causes a very sour taste,—a sense of burning along the throat and gullet, in the act of swallowing,—acute burning pain in the stomach immediately afterwards,—then violent vomiting,—next sudden failure of the pulse and strength,—and death in ten, twenty, thirty, or sixty minutes, sometimes under a state of pure and rapidly increasing faintness, sometimes at the close of one or more attacks of violent tetanic spasm. Such a succession of symptoms, within such an interval, cannot be caused by any other poison, or by any natural disease or combination of diseases, with which I am acquainted.

Strychnia is another poison which causes almost invariably symptoms quite characteristic of its action. If, within a few minutes after taking an intensely bitter substance, a person is attacked with pure and violent tetanus, recurring in frequent fits, and proving fatal in five, ten, twenty, or sixty minutes, I cannot conceive it possible to draw any other inference, than that death has arisen from poisoning with strychnia, or some of the poisons which contain it. These symptoms are almost invariable, at least in the case of strychnia itself. There is a way, indeed, in which this alkaline principle might be administered, so as to act diffe-rently, and to render it difficult to form the foregoing opinion. For obvious reasons, I shall not mention it. It can only be tried with success by one very well acquainted with the properties of the poison. On the whole, therefore, a decided opinion may be drawn in almost every instance from symptoms only; and this is a point of consequence, because other medical evidence will seldom be attainable, and nothing but the difficulty of procuring the drug keeps it now out of the hands of the poisoner.

" Corrosive sublimate is one of the poisons that produce characteristic symptoms only on some occasions. Several cases are on record, in which it has caused a strong metallic and astringent taste,—a sense of corrosion and burning in the throat or gullet, or both, in the act of swallowing,—acute burning pain in the stomach and belly soon afterwards,—speedy, violent, and often bloody vomiting,—afterwards purging of the same description; and, on the second or third day, or a little later, more or less salivation, with fetor of the breath, ulcers of the gums, dropping out of the teeth, and even gangrene of the mouth: all the signs, in short, of true mercurial salivation. In the event of such a case occurring in a criminal court, I cannot see how a witness could avoid giving his opinion that corrosive sublimate, or some soluble salt of mercury, had been taken.

"I shall now close this subject, by endeavouring to show that arsenic is another poison which may at times produce symptoms such as cannot originate in any other cause.

"Arsenic, when it does not prove fatal, or only after a week or upwards, often causes a singular complexity of symptoms, denoting inflammation, or at least violent irritation, in the throat, gullet, stomach, and intestines,—in the windpipe,—in the mucous membrane of the eyes and nose,—in the urinary bladder, urethra, and vagina: in short, in the whole mucous surfaces of the body. In such cases, too, there are occasionally eruptions of the skin,—sometimes petechial, sometimes measly, sometimes miliary: and more frequently the inflammatory symptoms are accompanied or succeeded, on the one hand, by complete or incomplete palsy of one or more of the extremities, and sometimes, too, racking pains in the palsied parts; or, on the other hand, by frequent fits of epilepsy, or by both together. No medical jurist could doubt that arsenic had been given, if he met with such a conjunction of disorders. They cannot be produced by any other poison, so far as our knowledge goes; and as little can they be caused by any natural disease, or any union of diseases ever known. It is true that such a union of symptoms from natural causes is conceivable; but, if it is so, it could never take place without its origin being clearly pointed out by collateral circumstances." (P. 318.)

*Observations on Cutaneous Absorption, with Experiments,*
by Dr. Dill.

The author thinks it very difficult to account for the unnatural secretion of urine which takes place in some conditions of the body, on any other principle than that of cutaneous absorption; and, with a view of establishing the existence of this, he has performed a set of experiments: these consisted principally in weighing persons very accurately, immersing them in baths, and again weighing them when taken out. Thus—

"A young man, weighing exactly 142 pounds, 5 ounces, and 2 drachms, whose pulse was 71, and heat 93°, descended into a bath, the temperature of which was 86°. After remaining in it for half an hour, and being again weighed, he had gained half a drachm. His pulse and heat during the experiment were unaffected. The expenditure which his body was hourly sustaining before the experiment, was two ounces and a half in the hour. When we, therefore, add ten drachms for the constitutional waste which he suffered in the bath during the half-hour of immersion, to that gained by the experiment, the amount of gain will be ten drachms and a half." (P. 354.)

To us it appears that the phenomena may be very satisfactorily accounted for, on the supposition that the contact of the water prevented the usual exhalation from the skin; while the thirty grains he had gained was probably a portion of fluid remaining upon different parts of the body. Dr. Dill, however, draws the following conclusions, from this and other experiments:

"In the first place, that the body in general increases in weight while in the bath; secondly, that, when its weight is unaffected by immersion, something must have been absorbed by it, to answer the natural expenditure of the system, which has been apparently suspended during the experiment; thirdly, that, when its weight is diminished, this arises either from an inactive condition of the cutaneous absorbents, or from the temperature of the bath being so high as to accelerate the action of the heart and arteries, and thus increase the perspiration; fourthly, that the additional weight acquired during immersion cannot be accounted for, either on the supposition of an increased pulmonary inhalation, or of a diminished evacuation by the skin; and, lastly, that the most simple and satisfactory method of accounting for this increase of weight, is by admitting the doctrine of cutaneous absorption." (P. 363.)

There follow a paper by Dr. ANDERSON, of Trinidad, in which baths and enemata of infusion of tobacco are strongly recommended in *Tetanus;* and some observations on the *Dysentery of Madeira,* by Dr. RENTON of that place, in which the author speaks highly of the mercurial plan of treatment. Neither communication is of much interest.

---

*Letters, Physiological and Moral, upon Animal Magnetism; containing a critical Exposition of the most recent Experiments; with a new Theory of the Causes, the Phenomena, and the Application of Animal Magnetism to Medicine.* By J. A. DUPAU, &c. &c. &c.—8vo. pp. 248. Paris, 1826.

DECIDEDLY sceptical as we have at all times been respecting the marvellous effects of animal magnetism, we are too well convinced of the powers of the mind over the body, either in a state of health or of disease, to doubt that the tricks of charlatans may have produced very striking effects; particularly when they have had to act upon the imagination of those who were feeble in mind, or morbidly sensitive in body. To admit, however, that the imagination may thus have been worked upon in some cases, is not sufficient for the true disciples of the "art." Either from a wish to impose upon others, or from an extent of credulity, to which perhaps the term of "monomania" might be not inaptly applied, they reject with indignation the explanation which we now offer, and contend that the effects produced, arise from the specific action of a magnetic fluid, transmitted at will from one person to another. It is not to be wondered at that the magnetising gentry should defend this point with determined obstinacy. Without this particular fluid, their "occupation's gone;" for the reality of animal magnetism entirely depends upon its existence.

The author of the volume before us is the editor of the Revue Medicale, and he has lately published several papers in that Journal upon the subject, of which he more formally treats in the work placed at the head of this article. He has even added something to the character of the "science of animal magnetism," by delivering some public lectures upon it.

The book consists of sixteen letters, addressed to ALIBERT. We shall pass them briefly in review, and give our readers the substance of their contents. It may be necessary to do our author the justice of premising, before we start with him as his companions in this magnetic investigation, that he is by no means the dupe or the disciple of the modern Magi. He acts, in fact, upon the principle of the motto he has chosen. He examines before he believes, that he may neither deceive others, nor submit to deception himself. The opening passage of the first Letter, at least, promises a rational examination of the subject.

"You ask me (says the author,) my opinion of animal magnetism, which some physicians are inclined to take into their serious consideration. To be sincere, I consider it a fantastic art, the mysterious proceedings of which have power only over men of morbid mind. It envelops in the same veil of error its propagators and its dupes. It is, in fact, a science, false in its theories and dangerous in its practice. Such is the result of my observations upon the subject. In endeavouring to unravel the disputes connected with animal magnetism, and to discuss with you its phenomena, I hope to destroy all that has been invented by credulity and ignorance to prove its power. It cannot be doubted that there is in every thing which is mysterious an unaccountable power, which acts upon us in spite of ourselves,—which leads us on, and subjugates us, in opposition to our better reason,—which fascinates our understanding, and disposes it to be the prey of every seduction and of every surprise. Such is the first source of the influence of animal magnetism."

By the unscientific and incorrect term of animal magnetism, is meant to be implied that concealed agent, discovered (or rather fancied to have been discovered) by the famous Mesmer, and which his partisans have made the principle of the phenomenon called magnetic. The expression, however, we may observe, is not of modern origin: it was employed by Van Helmont and Paracelsus, to designate certain modes of treatment considered useful in wounds, and in some particular diseases.

We shall not follow our author through the "philosophical history of animal magnetism." It may be sufficient to observe, that, from the most ancient periods, certain arts have been had recourse to, for the furtherance of interested views, and the support of particular doctrines, either of philosophy or religion. The same key which unlocks the mysteries of the ancient temples, and the magic machinations of the middle ages, will also throw open to our view the physical and moral phenomena of animal magnetism. It is well known that the Egyptians, who extended so far every useful art and science, also raised to perfection the power of acting upon the moral and physical constitution of man by various fascinations. Their priests, who were their first physicians, well knew the influence which might be produced upon certain diseases by excitement of the mind. The sight of consecrated objects, mysterious ceremonies, and a variety of other contrivances, were had recourse to, and with the effect of producing very remarkable impressions upon excitable and credulous imaginations. The influence of the Divinity was supposed to be exerted. How skilful the Egyptian priests were in working upon the imagination of their victims, and in exciting the nervous system, may easily be imagined by the convulsive paroxysms and the mental extasies of those who were newly initiated. Numerous were the means these magi kept carefully concealed within the interior of their temples, to maintain their mighty influence over the people. That they possessed a considerable fund of useful information, is not to be doubted. It was all, however, subservient to their master-passion,—to the ambition of securing the mental slavery of the multitude. In some Egyptian monuments which are still preserved, may be seen the magnetising priest at the bedside of the patient. The attitude of Anubis is precisely that of the modern magnetiser. To the Egyptian priests we are also indebted for different amulets, and many other magic means, which at once imposed upon and cured their credulous countrymen. The mention of these circumstances alone will be sufficient to draw the attention of our readers to the analogy we wish to trace between the ancient mysteries and the practice of the modern disciples of animal magnetism.

We know not the proceedings by which the Grecian priests succeeded in elevating their priestesses to that pitch of mental excitement denominated "divine fury," but the similarity of the effects to those arising, under certain circumstances, from animal magnetism, appears sufficiently evident. These pious frauds would have been pardonable, if they had been confined to the treatment of disease. Such, however, was not the case. The influence which one set of men possessed over the majority, was turned to the most debasing purposes. The people were fettered, both in mind and body, by spells of enchantment. We doubt not but that the magnetising conjurers of the present day would like to riot in the spoils, which their brethren of old so well knew how to wrest from those whom they held in bondage.

The modern magnetisers would wish us to believe that at least they have the merit of proposing to us a new system. Our author, however, does not fail to prove the contrary: he quotes a passage from Plautus, which completely settles the point. Mercury, having assumed the figure of Sosia, doubts whether he shall free himself from his presence, by inflicting a few well-applied blows, or by putting him to sleep. "Quid (says Mercury,) si ego illum tractim tangam ut dormiat." The practice, then, of animal magnetism to procure sleep possesses no charm of novelty. We would refer those who wish to be convinced of the antiquity of this system of quackery, to the Dictionnaire des Sciences Medicales, art. *Magnétisme Animal,* tome xxix. p. 463. The article is elaborate and interesting, containing a detailed account of this pretended science.

It has been well observed by Buffon, that, when the human mind is struck by any singular object, much gratification is derived from rendering it still more extraordinary, and in attributing to it chimerical and marvellous powers. Hence we find that patients have frequently been cured by the touch,—by certain signs,—by tracing a wand over the body,—by being placed in a variety of ridiculous attitudes, and by other means equally absurd. We cannot venture to say that the ancient astrologers removed many diseases by their celestial treatment; but, in common with the magnetisers of the present day,—in common with all those who are aware of the effects that may be produced by excitement of the nervous system and the imagination,—they sometimes produced real cures. It would appear that at all times the regular and rational physicians have been opposed to these charlatans.

The overwhelming influence which particular sects have from time to time exerted over the passions and general conduct of the multitude, is next considered; and indeed requires no laboured evidence. It is equally true that, in numerous instances, the body has been acted upon through the medium of the mind, as the mind has through the body, and that diseases have sometimes been relieved, and sometimes been caused, in this manner. It is necessary, however, to bear in mind the close affinity between the sectarians of a former age and the magnetisers of the present moment, both with respect to the mysticism of their dogmas, and the various stratagems to which they have recourse. It may ruffle the temper of the true believers of the "specific magnetic fluid" of modern date, thus to trace the origin of their mummery, and to reduce their practices to their proper level: the statements may be unpalatable, but for our part we have no doubt of their truth. The extraordinary scenes that occurred at Paris during the time of the *convulsionists* of St. Médard, were lamentable proofs of how much might be effected upon weak imaginations by the fraud or insanity of a few. We must refer to the work itself for a detailed description of these pitiable follies. The moral epidemic lasted for some years, until at length a *jeu de mot* put an end to the affair. The following distich was written over the door of the cemetery:

> "Il est défendu de par Dieu
> De faire miracle en ce lieu."

The next Letter discusses the progress of animal magnetism since the time of Mesmer. The first ideas of the art were not suggested to this idle speculator by the direct observation of any phenomena which the mutual relations and influences of men presented, but by a false theory of the universality of a magnetic fluid, which established the harmony of the celestial bodies, and the emanations of which acted upon the human frame. We cannot pass through the labyrinth of errors which owed their origin to Mesmer. His reputation was of short duration, although for some time he turned his jugglery to good account.

The subjects next treated of are "the different Theories of the Magnetisers; the Magnetic Fluid; the Will."

"It is a very general remark, (says the author,) and one which may be applied to every new kind of knowledge, and to all researches which become the objects of discussion, that, when at different periods the attention of mankind is directed to the same object,—when people of different countries, and at distant epochs, occupy themselves zealously in its investigation,—we may be assured that there is some foundation for such novelties, and that they offer some positive results. But the sum of error is frequently so considerable, that it is difficult to separate the truth from the falsehoods in which it is disguised."

Such is the case with respect to animal magnetism, employing the term in its modern acceptation. Extraordinary effects have no doubt been produced, but the interested and pretended adepts in the art have refused to admit a reasonable explanation of them.

"The first idea which led to the use of the term *magnetism*, was the affinity which it was thought was detected between the properties of the mineral loadstone, and the reciprocal action of two individuals. But what kind of similitude can there exist between the power of attracting iron, of taking a direction towards the poles, &c. and that which a magnetisor exercises by his fascinations upon a credulous and diseased individual."

Our author, notwithstanding the castigation he very properly bestows upon the trickeries of former times, is inclined to be somewhat more charitable to the modern magnetisers. He forgets, probably, the relationship he has all along endeavoured to establish between the actors in both the ancient and modern farces, when he says that "he does not suspect the magnetisers of *mauvaise foi*." He is only willing, however, to grant them honesty at the expence of understanding: the terms are hard, but they are precisely those which we should ourselves have imposed.

In the Letter which describes the "Management of Animal Magnetism," we find that either the imagination or the nervous system must be acted upon. In the magnetiser himself, these may remain perfectly passive, and, provided he is capable of adroitly acting either upon the one or the other in a person predisposed to such a species of affection, he will produce those variable phenomena termed magnetic. M. Rostan, the author of a singular article upon this subject in the Dictionnaire de Medécine, vol. xiii. requires that the magnetic operator should be in good health,—that he should have nothing repulsive about him,—that he should be in the prime of life,—that he should be superior to the person magnetised,—that he should appear to be animated by a spirit of benevolence,—and, lastly, that *a man should be employed to magnetise a woman*, and *vice versa!* We may observe, en passant, that pregnancy is not an uncommon effect of the art!

It is worthy of particular notice, that the most bigotted admirers of this science, as it is called, are compelled to admit that they exert their most skilful efforts in vain upon healthy persons who have no prepossessions in favour of their avocation. In other words, their proselytes are limited to those who are foolish by nature, or infirm from disease.

Passing over any formal enumeration of the magnetic phenomena, which may easily be imagined in all their variety, we come to the eleventh Letter, and must venture to waste a sentence or two upon the strong hold of the true believers in magnetic potency.

"Somnambulism, in all its varieties, has been considered, since the period of M. de Puységur, to afford the most positive evidence of magnetic influence. A magnetiser, with an imposing attitude, a fixed look, stretches forth his hands over his patient, whose body and mind are perhaps enfeebled by suffering, and, after having gained full possession of his imagination, he fatigues his senses by monotonous gestures. The patient gradually falls asleep."

There is here no mystery; the effect might be anticipated: the magnetic fluid is not required. Upon the same principle a child is lulled to rest by fatiguing its senses with some nursery lullaby, or some gentle and oft-repeated motion. That many cases of somnambulism, in the strict definition of the term, have occurred, is very certain; and it is only where persons have been disposed to this kind of imperfect sleep, that the magnetisers have apparently succeeded. They first fatigue the senses, and frequently produce a high state of cerebral excitement, which will be in proportion to the particular idiosyncrasy upon which they have to act, and sleep may follow; but, as it is artificially provoked, and connected with a variety of moral impressions, it is not to be wondered at that somnambulism is the frequent result.

It appears, however, the magnetisers assert that the "magnetic somnambulism" differs from that which occurs from other causes. The following are the stated distinctions: —There exists between the magnetiser and the somnambulist an intimate relation, so that they communicate with, understand and answer each other; whilst the latter is completely isolated from other persons and from external objects. As usual, however, when their part was to be acted in the presence of unbelievers in their necromantic skill, "*le magnétisme fut sans effet, et les magnétiseurs presque confondus.*"

The last part of the subject we shall notice is "the application of animal magnetism in the cure of Disease."

In many parts of the continent, the power of animal magnetism is spoken of with much enthusiasm in various diseases. We have already admitted the possibility of very imposing effects being produced by such means, and have offered what appears to us a rational explanation of them. It may be observed, that it is only in derangements of the nervous system that the most zealous advocates promise any benefit from animal magnetism; and it certainly did not require any particular proof from them, or any exercise of their art, to establish that which nobody will deny,—that the body is frequently, and in various ways, affected through the agency of the mind. The effects of the well known and justly ridiculed *piperies* of Hohénlohe, and his cures, so much vaunted by the fanatics who have yielded up their senses to his assertions, are to be explained in the same manner.

It is with infinite regret we find the name of Rostan coupled with an absurd tale of a young woman seeing with the back of her neck. Is it possible, after reading his account (in the Revue Medicale), that we can approach the labours of this author, on other subjects, without doubting either the soundness of his judgment or the good faith of his statements? We are at all times unwilling to hazard so serious an accusation as that of deliberate misrepresentation, and we will not do it even on the present occasion. Our readers must determine for themselves. But we ask, if M. Rostan and his friend believed that the subject of their experiment, the *magnetised somnambulist,* could tell the hour at which the hands of a watch were placed, when it was close to the back of her neck, as they assert, why was she not exhibited in the presence of the persons who were at the moment anxious to ascertain the truth of the various statements that were made upon the subject? Why was this most extraordinary case not published until all the means of verifying it were lost? To answer a man sincerely, and at the same time civilly, who asserts "I have seen the thing," is often difficult. We would be inclined to adopt the reply of Fontenelle to the wonderful observations of the philosopher—"*Vous l'avez vu, je le crois; mais si je l'avais vu, j'en douterais.*"

Such is the influence that the magnetic operators are said by M. Rostan to gain over their proselytes, that they follow them "*comme un chien suit son maitre.*"

The discussion which has lately taken place at the Académie Royale de Medécine upon the subject, is given in the concluding Letter. Like most debates in medical societies, (although we believe many useful hints are thus frequently suggested, and professional zeal kept up,) the argument terminated, after a little amicable wrangling, by a decision that nothing had been decided.

We have stated our belief that animal magnetism is a mere system of juggling. Such also is, to a great extent, the opinion of the author whose work we have just noticed. So long, however, as pretended philosophers and poverty-stricken practitioners are to be met with, who are infected with the *auri fames,* so long will efforts be occasionally made to impose upon the credulity of human nature. Had not the subject of animal magnetism at present excited the serious consideration and examination of many respectable men and learned societies upon the continent, we should hardly have ventured to obtrude the subject at all upon the notice of our readers. It is our duty, however, to record not only the practical improvements, but likewise to take an occasional glance at the passing follies, of the day.

## Figures and Tables

**Figure f1:**